# Embodied understanding

**DOI:** 10.3389/fpsyg.2015.00875

**Published:** 2015-06-29

**Authors:** Mark Johnson

**Affiliations:** Department of Philosophy, University of Oregon, Eugene, OR, USA

**Keywords:** understanding, embodiment, embodied cognition, meaning, emotion, bio-functional understanding

## Abstract

Western culture has inherited a view of understanding as an intellectual cognitive operation of grasping of concepts and their relations. However, cognitive science research has shown that this received intellectualist conception is substantially out of touch with how humans actually make and experience meaning. The view emerging from the mind sciences recognizes that understanding is profoundly embodied, insofar as our conceptualization and reasoning recruit sensory, motor, and affective patterns and processes to structure our understanding of, and engagement with, our world. A psychologically realistic account of understanding must begin with the patterns of ongoing interaction between an organism and its physical and cultural environments and must include both our emotional responses to changes in our body and environment, and also the actions by which we continuously transform our experience. Consequently, embodied understanding is not merely a conceptual/propositional activity of thought, but rather constitutes our most basic way of being in, and engaging with, our surroundings in a deep visceral manner.

## Introduction

The term “biofunctional understanding” was introduced by Iran-Nejad in a technical report in 1980 and subsequently elaborated in a number of publications (e.g., Iran-Nejad and Ortony, 1984; Iran-Nejad, 2000; Iran-Nejad and Stewart, 2011; Iran-Nejad and Bordbar, 2013). It is an especially apt expression for the way we should approach a theory of human understanding. It reminds us that we need to see how our capacities for understanding and reasoning are grounded in biological processes of organism-environment interaction. The “bio” component refers to the fact that we are biological organisms evolved both to sustain in our person the conditions of life and to enhance its quality. The “functional” component refers to the fact that our activities as biological organisms give rise to our capacity to perform a wide range of cognitive and affective functions. These specific emerging functions define who and what we are, and they shape the way we make sense of our experience, including all of our higher order cognitive operations. These life-sustaining and life-enhancing functions are embodied in the sense that there is no mind, thought, valuing, or action that is not in some way dependent upon our bodily makeup and patterns of engagement with our world. In short, our very ability to understand our world and other people arises from the nature of our bodily existence (including both our physical body and the structures and processes of our brains) plus the embodied interactions we have with our material and cultural environments.

In this article, I shall attempt to explain how our bodily nature and activities give rise to understanding, by which I mean our way of situating ourselves more or less successfully in the world, in a manner that allows us to make sense of our surroundings, ourselves, other people, and cultural institutions and practices. This embodied orientation challenges the unsatisfactory notion of understanding as merely an abstract intellectual grasping of concepts and their relations. I argue, instead, that understanding is a full-bodied, full-blooded, fully passionate process that reaches down into the visceral depths of our incarnate experience and connects us functionally to our physical-cultural world.

## Our Inherited Conception of Understanding as Disembodied

The chief obstacle to employing the phrase “embodiment of understanding” is that the dominant intellectual traditions within Western culture have bequeathed to us a view of understanding as either completely disembodied, or at least not primarily dependent on the nature of our bodies and brains when it comes to the structures and processes of our conceptualization and reasoning. I begin, therefore, with a brief account of this received folk theory of relatively disembodied understanding that stands in the way of a deeper and more adequate appreciation of how the body grounds and participates in mind. Although I have several times (Johnson, 1987, 2007, 2014; Lakoff and Johnson, 1999) given strong critiques of what I have called “disembodied” views of mind, thought, and language, I have sometimes thought that this is perhaps too strong a term for the views I am challenging, since only the most extreme ontological dualists think that mind can exist without a body. Instead, the view of disembodiment I am criticizing is the assumption that the structures of our concepts, understanding, and reason are not grounded in the nature of our brains and our sensory, motor, and affective capacities. Therefore, I regard any theory (including materialist theories of mind) that tries to explain meaning, understanding, and reasoning without detailed reference to the nature and workings of our bodies as “disembodied” in the broad sense.

Although there is in the West a long history of disembodied views of mind, thought, and language, for our purposes we need only trace the history back to the Enlightenment faculty psychology that has so fatefully shaped our commonsense and theoretical views of mind down to the present day. The basic idea of faculty psychology is that any achievement or operation we attribute to creatures with minds can be explained in terms of the activity of discrete powers or capacities (i.e., faculties) that either individually or conjointly produce the various kinds of judgments of which mind is capable. Each faculty supposedly has its own distinct nature (essence) by virtue of which it carries out certain specific functions, such as perceiving, feeling, forming concepts, reasoning, judging, or willing. For example, our ability to perceive objects allegedly depends upon our capacity to passively receive sense impressions (of colors, textures, odors, sounds) of objects in the world. This alleged *faculty of sensation* is typically thought to cooperate with *the faculty of imagination*, by which we order sensations into unified images or representations that can persist over time. According to most faculty psychologies, full-blown knowledge of an object additionally requires a *faculty of understanding* that supplies concepts for thinking (i.e., conceptualizing) the object that has been presented to our senses and ordered by us into a unified image. Finally, we are supposed to be able to then expand our knowledge by using our *faculty of reason* to connect, according to the laws of logic, propositions into larger units (systems) of knowledge. Faculty psychology strikes most people in Western cultures as simple common sense, even though it is an artifact of centuries of philosophical theories of mind, thought, and knowledge.

Hobbes, Locke, Berkeley, and Hume all had influential faculty theories of mind and thought, but Kant’s famous account has no doubt had the greatest influence on our current perspective. To oversimplify a bit (though not unfairly), Kant thought of understanding as the power to create concepts and to apply them to sense contents or other representations to generate knowledge of objects of experience. He regarded understanding as an activity of judgment, where “all judgments are functions of unity among our representations” (Kant, 1781, A69/B93-94). Understanding, in Kant’s theory, performs unifying judgments that combine one or more representations under some concept. He explains: “... concepts rest on functions. By “function” I mean the unity of the act of bringing various representations under one common representation” (Kant, 1781, *A68/B93*). Concepts are thus formal rules for ordering (i.e., making determinate in thought) various images or other concepts. For instance, according to Kant’s view, the concept *chair* would be a rule (of the faculty of understanding) specifying the features that together constitute a given object as a chair, and in this way the concept supposedly unifies a number of sense impressions into an object of knowledge (e.g., a chair). More general concepts can also unify subsidiary concepts, such as when the concept *furniture* contains within it concepts for chairs, tables, beds, sofas, etc. Concepts, for Kant, are thus formal structures, insofar as they identify the necessary and sufficient properties or features something must have to be *that* particular kind of thing. Kant succinctly summarizes the relative contribution of both intuitions (as the product of the faculty of sensations) and concepts (as the product of the faculty of understanding): “Our knowledge springs from two fundamental sources of the mind; the first is the capacity of receiving representations (receptivity for impressions), the second is the power of knowing an object through these representations (spontaneity in the production of concepts).... Intuition and concepts constitute, therefore, the elements of all our knowledge, so that neither concepts without an intuition in some way corresponding to them, nor intuition without concepts, can yield knowledge” (Kant, 1781, A50/B74).

As the past two centuries of Kant-influenced philosophy and its critics have demonstrated, once you distinguish the matter of sensations from the formal structure of concepts, you cannot really explain how the two get combined in acts of judgment. Once Humpty-Dumpty is broken (i.e., once form (the concept) and matter (the sensations) are separated), you cannot put Humpty back together again. In his famous “Schematism of the Pure Concepts of Understanding” (Kant, 1781, A137/B176—A147/B186), Kant attempted to bridge the gap between the material of sensations supplied by the body and the formal structuring supplied by the mind by finding a third capacity—imagination—that supposedly has one foot in the material and another in the formal, and somehow unites them in one synthetic act. However, as has been well documented in Kant scholarship, this unifying move leaves the faculty of imagination a bit out in no man’s land. On the one hand, it seems bodily in the way it constitutes images out of sensations. On the other hand, it remains non-bodily in the way it generates formal schemata (Johnson, 1987). This indeterminate status for imagination shows up in the *Critique of Pure Reason*, where Kant sometimes aligns imagination with sensing and our bodily formation of images and other times with understanding and its capacity for spontaneously generating synthesizing forms.

I cannot discuss the intricacies and problems of Kant’s schematism here. It is enough for our purposes to recognize that understanding (*Verstand*) has been defined as a faculty of concepts and conceptual unifying judgments, in contrast with the contribution to knowledge made by the bodily processes of sense perception and imagination. Concepts are the products of the synthesizing power of the mind that allows us to grasp the form of objects of knowledge. Kant insists that reason has an a priori structure that makes possible logical relations and logical inference that are supposedly in no way dependent for their structure on the bodily makeup or experience of any reasoning being.

Kant was not a Cartesian substance dualist (where “mind” and “body” are two different kinds of substance); rather, he has a dualism that aligns sensing and feeling with the body, and conceptualizing and reasoning with acts of a transcendent ego, which is the source of a spontaneous organizing activity.

This “disembodied” view of understanding has seemed just right to many so-called functionalist philosophers of mind, since they regard mental operations as functional programs for manipulating representations based only on their formal (syntactic) properties. Kant’s view of determinate judgment as a synthetic operation through which concepts and other representations are combined into propositional judgments having a subject-predicate structure perfectly fit the information processing view of mind that arose in the middle of the last century. On this view, sentences in natural languages are taken to express subject-predicate propositions that can map onto mind-independent aspects of the world, thus generating objective knowledge of the world. Contemporary functionalist theories of mind (such as Fodor’s (1981) program and the view Hilary Putnam once held (Putnam, 1967) and later rejected) are good examples of relatively disembodied views of mind. When Putnam (1981, 1988) eventually came to reject his early functionalism, it was precisely because it lacked the requisite bodily engagement with the environment that is necessary for any understanding and knowledge of the world.

## The Embodiment of Understanding

To say that understanding is embodied requires a fundamentally different orientation toward mind, thought, and language that contrasts markedly with our received view of understanding as an intellectual, disembodied, mostly conceptual process. Embodied Cognition views start with the recognition that the locus of all our human perception, meaning-making, valuing, thought, and action is a series on ongoing organism-environment interactions (Dewey, 1925). Humans are embodied creatures that, before any other cognitive activities are possible, must first maintain the requisite conditions for their bodily integrity and life maintenance. Understanding is not just an intellectual operation, but rather a series of full-bodied engagements with significant aspects of one’s environment that are meaningful for them and that sustain their life and growth, and this happens not just at the physical level, but also at the interpersonal and cultural levels. In other words, understanding is our way of making sense of and inhabiting the world in which we live, so that we can go forward with our lives. Understanding is thus less a form of *knowing or thinking* than it is a matter of *experiencing and acting*. To say that we *understand* something is to say that we grasp its meaning in a way that allows us to be at least somewhat “at home in” and not alienated from our world. “Grasping” meaning is not restricted to an intellectual act, but is rather a process of intelligent bodily organism-environment interaction. This “intelligence” refers to our whole embodied engagement with our surroundings, and not merely to some intellectual operation of thought. We “understand” some object, event, or idea when we grasp its significance for past, present, and future activity and are able to carry that understanding forward into new experience. Therefore, understanding is a form of embodied adaptive and transformative experience, since developing a new understanding actually remakes experience.

Let us consider in more detail and depth the steps of the argument in the previous paragraph. To develop an adequate understanding of experience, the starting point must be organism-environment coupling through which both organism and environment change over time and establish new relations. In order to have a viable organism, you need a body—that is, a living physical bodily structure with an interior and an exterior that are demarcated by a semi-permeable boundary that allows the organism to take in energy, excrete waste, and operate with relative integrity. Neuroscientist Antonio Damasio nicely summarizes the conditions for an organism’s management of its life functions in relation to its surroundings:

(L)ife requires that the body maintain a collection of parameter *ranges* at all costs for literally dozens of components in its dynamic interior. All the management operations to which I alluded earlier—procuring energy sources, incorporating and transforming energy products, and so forth—aim at maintaining the chemical parameters of a body’s interior (internal milieu) within the magic range compatible with life. The magic range is known as *homeostatic*, and the process of achieving this balanced state is called *homeostasis*. (Damasio, 2010, p. 42)

This grounding in organism/environment interactions means that, as both James (1890) and Dewey (1925) argued, we must not base our account of understanding on any abstractions from experience, such as “sensations,” “concepts,” “propositions,” or “knowledge judgments.” To cite just one example, we must avoid the classical empiricist error of assuming that all experience begins with atomistic sense impressions that have to be synthesized, via associative processes, into unified perceptual judgments. Contrast this erroneous view with the fact that what we actually experience are whole, unified situations, within which we experience individual objects, qualities, and events through the selective discriminations by which we individuate aspects of our experience. If we keep in mind that experience includes both the structure and activity of the organism as well as the structure of the environment, we will appreciate how bodily processes are absolutely crucial for the possibility of any form of cognition, feeling, or action, and we will not abstract away from the body and its environments. Otherwise, we end up selecting some part or phase of an experience and then mistakenly assuming that what we have selected out (a sensation, quality, concept, image, judgment) defines the whole of that experience, in all its depth and richness.

The second important point is that the homeostasis necessary for life should be thought of as what Jay Schulkin (2011) calls allostasis—a dynamic equilibrium in which the organism can, as it interacts with its environment in an ongoing fashion, establish a new equilibrium set-point, and does not merely return to some pre-established, fixed set-point. According to Luu and Tucker (2003) “allostasis can be defined as the regulation of many variables (including behavioral as well as physiological variables) over time to meet motive set-points that are established dynamically... Rather than just responding to deviations from fixed set-points, allostasis allows the organism to anticipate needs and to adjust configurations of regulatory goals in advance to meet those needs” (Luu and Tucker, 2003, pp. 125–126). It is only within a bounded organism that homeostasis or allostasis are possible, so, once again, we must keep our focus on the nature and conditions of the embodied organism in its many environments.

The third key point is that it is always within the context of organism-environment transactions that values arise and influence behavior. Our experience is value-laden all the way down to the primitive emergence of the values required for life maintenance. Damasio concludes, “I see value as indelibly tied to need, and need as tied to life. The valuations we establish in everyday social and cultural activities have a direct and indirect connection with homeostasis.... Value relates directly or indirectly to survival. In the case of humans in particular, value also relates to the *quality* of that survival in the form of *well-being*” (Damasio, 2010, pp. 47–48). This last sentence is crucial, insofar as it recognizes that, for creatures as complex and interrelated as we are, our primitive values include not just maintenance of bodily equilibrium necessary for life, but also maintenance of social and cultural equilibrium within larger communities.

Because the focus of my argument is not directly on values, I will not elaborate Damasio’s extensive and nuanced treatment of the origin and development of human values (see Damasio, 2003). I emphasize the pervasiveness of value only to emphasize that understanding is irreducibly value-laden and tied to our specific environments, both physical and social/cultural. Understanding is not just a matter of concepts and propositions capturing some aspect of the world; rather, it is a matter of appropriately situating and enabling purposive action in the world, relative to our well-being.

I also want to highlight the way in which values first emerge from our bodily, interpersonal, and communal needs, which are realized primarily in and through our bodily activities. In short, “the values that humans attribute to objects and activities would bear some relation, no matter how indirect or remote, to the two following conditions: first, the general maintenance of living tissue within the homeostatic range suitable to its current context; second, the particular regulation required for the process to operate within the sector of the homeostatic range associated with well-being relative to the current context” (Damasio, 2010, p. 49). Note that the “current context” referred to here would typically be not merely our physical surroundings, but equally our interpersonal and communal relations.

## Emotional Dimensions of Understanding

The next (i.e., fourth) characteristic of our embodied understanding is so important that it deserves its own separate heading. The tendency to regard understanding as an intellectual, cognitive, conceptual, or propositional activity has led to the downplaying or even denial of any role for emotion and feeling. On the face of it, such a separation is ludicrous, but that has not stopped committed cognitivists from excluding emotions from the domain of understanding, or even from regarding emotions as contrary to acts of understanding. Nothing could be farther from the truth.

To see immediately why this dismissal of emotion is such a devastating mistake, we need a brief consideration of the nature and function of emotions in humans (and other animals). Here again, Damasio’s work has been quite influential, based on his extensive research on the role of emotions and feelings in the formation of self, the emergence of consciousness, and the nature of our values. The whole story of emotions, feelings, and their neural and chemical substrates is quite long and detailed, but a few key points are relevant to the argument I am making here.

Put simply, emotions are neural-chemical-bodily response patterns resulting from an organism’s monitoring of its changing body-state as it interacts with things in its environment. These responses are generated—mostly unconsciously and automatically—when the organism detects some “emotionally competent stimulus” (Damasio, 2003, p. 53) that the organism registers as requiring some change in its body-state. Most of these response patterns for the basic emotions have been established and sedimented over our evolutionary history. “The ultimate result of the responses, directly or indirectly, is the placement of the organism in circumstances conducive to survival and well-being” (Damasio, 2003, p. 53). Consequently, emotions are the result of our most important capacities to appraise our situation in order to act appropriately within it. Most emotional response patterns are automatic and operate beneath our reflective awareness, but sometimes they are also accompanied with feelings of those emotions, in which cases we are able to be consciously aware of the felt changes in our body-state as it engages its environments. In short, emotional response patterns constitute the body’s (mostly unconscious) registering of how it is being altered by its engagement with its surroundings, and we *feel* an emotional state whenever we become conscious of those changes in our body state. So, we can be in an emotional state without necessarily being aware (through feeling) that we are in that specific state. That is why, for example, we can *be* angry, even before we *feel* angry.

Emotional response patterns and emotional feelings thus lie at the heart of our capacity to *understand* the various situations in which we find ourselves. Emotional response patterns emerge because of their general, though not always complete, suitability for helping us survive and enhance the quality of our existence. Consequently, our ability to understand what is happening in a developing situation, as well as figuring out what it means for us, is dependent on our emotional makeup.

Emotions are therefore an integral part of human understanding and meaning. I use the term “meaning” here in the way it was understood by the classical American Pragmatist philosophers. Basically, any thing (object, quality, event, person, idea) has meaning just insofar as it points to some experience, either past, present, or future (projected) that is for us connected with it. Things are meaningful because they afford us various possible experiences. For example, with respect to *past* experience, the term “gun” might have a complex meaning that recalls your experience with guns, the history of changing weapons technologies, knowledge of your culture’s use of and attitude toward guns, and the history of gun violence, both at home and abroad. In your *present* experience, the meaning is tied to certain objects (guns) that you can see, handle, and use in various ways. This would include our visual experience of guns and our motor programs for using guns. In relation to our projected *future*, *gun* might afford various possibilities for certain definite kinds of experiences, such as hunting, target practice, warfare, mass shootings, police practices, and criminal operations. This Pragmatist conception of meaning is not just tied to words in a language, for it stretches out to include any form of symbolic activity in which meaning can emerge (e.g., painting, sculpture, architecture, music, dance, spontaneous bodily gestures, ritual practices, etc.). This means that our understanding of guns will include a vast body of visual, tactile, auditory, gestural, movement, object manipulation, and other possible experiences.

Within such a broad notion of meaning, emotions can play a crucial role, because they direct us toward tendencies for past, present, and future experiences. Emotional response patterns indicate, at the deepest levels of our engagement with our world, the perceived values to us of things and activities and the tendencies of various qualities, objects, and events. Brentano (1874) argued that the mark of the mental is intentionality—the directedness of a mental state toward some object. Emotions exhibit intentionality just as much as linguistic terms and concepts do. Emotions point to and mark the character of various situations in which we find ourselves. My joy this morning is not merely an internal mental state locked within my body-mind, but rather it marks the character of my world as it stretches out before me and affords me various possible experiences and modes of activity and response. Instead of saying merely that *I am joyful*, it is more accurate to say that my *situation—my mode of being in the world—is joyful*.

It is in this broad sense that emotions are just as much a crucial part of understanding as concepts and propositions are. Emotions are one of our primary and most important ways of taking the measure of our situation. They are appraisal processes that help us to orient ourselves meaningfully within a certain context and to grasp various possibilities for meaning and action.

## Understanding Concrete Concepts

Those who are enamored of disembodied views of understanding will claim that embodiment views cannot adequately account for the full range of human conceptualization, reasoning, and language. While they may grant that structures of embodied meaning play a role in the semantics of ordinary concrete physical objects and events, they will insist that abstract concepts cannot be grounded in these embodied structures of meaning. So, the important question arises: How do we get from the dimensions of embodied understanding sketched above to our full capacity for abstract symbols and formal reasoning? The answer lies with the recruitment of sensory and motor capacities to perform conceptualization and rational inference.

Before considering abstract understanding, let us first consider the role of the body in how we understand the meaning of a simple physical object like a cup. Barsalou (1999) has pointed out that the meaning of concrete physical objects is not merely some abstract feature list of properties that supposedly define that kind of thing. He explains, “a concept is not a single abstracted representation for a category, but is instead a skill for constructing idiosyncratic representations tailored to the current needs of the situated action” (Barsalou, 2003, p. 521). The meaning of an object, and our conception of it, involves our simulation, via functional neuronal clusters involved with sensory, motor, and affective experiences, of various actual and possible interactions with the things we call *cups*. In other words, the meaning of any object would be the kinds of experiences we have had, are now having, or might someday have, with that sort of thing. For a cup, for example, some of these experiences would be visual—the various views we might have of different cups as we regard them from multiple perspectives. Most of those views would present the perceiver with a physical container structure that has a three dimensional boundary with an interior and an exterior, capable of holding liquids and solid substances. Some cups would have handles, others would not. Some would be delicate china, others thick and heavy, and still others thin and made of cheap materials (paper, plastic, etc.). Our concept of a cup is not limited merely to visual simulations, however. Our visual simulations of cups would be coupled with simulations of the tactile and motor interactions we have, or might have, with various kinds of cups. Neural imaging studies show that when we see a cup, we are not only having activations in the visual areas of the brain, but also activation of motor programs and tactile sensations. Seeing a cup will thus activate areas in the motor and pre-motor cortex that would be involved in touching and handling cups, even though we are not actually handling them (Gallese and Lakoff, 2005). These motor simulations would include operations such as the arm, hand, and finger movements that are necessary to grip cups of various types, sizes, and materials, and to raise them to our lips, take a drink, and return them to a table. Thus, there would be the motor synergies (activated in motor cortex in the brain) required to partially close each finger, with just the right force so that we do not either drop or break the cup. These hand and finger movements also need to be coordinated and sequenced into a fluid hand-closing gripping gesture that is just right for the size, location, and material makeup of the cup. This sequencing is done in the pre-motor cortex.

Our visual, tactile, auditory, and motor simulations of perceptions of, and interactions with, cups are still not the whole story. An adequate account would need to include what are traditionally called the “esthetic” dimensions of our experiences with cups. This would involve aspects such as our experiences with the different types of cups (e.g., fine china tea cups, mugs, paper cups, plastic cups) that figure in the meaning of *cup*. It would also need to include our sense of the social and cultural contexts in which these different types of cups are appropriately used (e.g., tea ceremonies, dinner parties, meetings in cafes, family meals, picnics, beer parties). These latter activities constitute what are known in cognitive linguistics as “frames,” which are complex event structures that have roles for agents, objects, actions, causation, purposes, outcomes, and so forth (Fillmore, 1982; Lakoff, 1987; Feldman, 2006).

Our understanding of something as ordinary as a cup actually turns out to be a fairly complex blending of simulated perceptions, emotions, and actions associated with the things we call cups, within specific social and cultural contexts. It involves bodily activities of perception of qualities, forms, spatial locations, internal structure, spatial relations, etc., and also activities of simulated body movement and object manipulation that are appropriate in various cultural settings. This embodied account of understanding and meaning has come to be known as Simulation Semantics. In his book, *Louder than Words: The New Science of how the Mind Makes Meaning* (Bergen, 2012), has presented scores of experiments showing how semantic simulation operates as we hear and read sentences and grasp their meaning. He calls his view the “embodied simulation hypothesis,” which asserts that “we understand language by simulating in our minds what it would be like to experience the things that the language describes” (Bergen, 2012, p. 13).

The crucial moral I draw from this research on simulation semantics is that understanding is *not* just an intellectual operation on disembodied concepts, ideas, or representations. Instead, understanding is a profoundly bodily process of experiential simulation that uses complexly interconnected brain regions responsible for all sorts of perceptual and motor activities, as well as emotional responses and feelings. It is critically important to keep in mind that the embodied simulations I have described here are typically not conscious reflective acts, but rather are carried out automatically and very rapidly, usually beneath the level of our conscious awareness. In other words, we do not actually have to consciously imagine that we are seeing or handling a cup, even though the functional neural ensembles required for these simulations are activated in our brains.

## Understanding Abstract Concepts

Up to this point, I have considered only our embodied understanding of the concrete physical object *cup*. But that is merely a small part of the larger story of human understanding, for we have to include some account of the bodily grounding of our understanding of abstract concepts like mind, knowledge, love, justice, fear, cooperation, etc. Explaining the possibility of abstract understanding is a vast project, so here I must be content to give a few examples of how parts of a comprehensive theory of the bodily basis of abstract understanding could be developed. Consider, for example, the abstract notion of a *state*, such as being hot, cold, weak, invigorated, in love, depressed, or in trouble. Notice that in English (and most languages) we speak of states as if they were metaphorical locations into and out of which some object, event, or person could move. Thus, we use the language of physical location and motion to metaphorically understand changes of state, as in “The water *went from* cold *to* hot in a matter of minutes,” “He fell *in* love,” “She clawed her way *out of* her depression,” “He keeps getting *into* trouble,” and “She *fell deep into* debt when her hedge fund collapsed.” (Lakoff and Johnson, 1980, 1999) observed that such conceptualizations of change of state involve a conceptual metaphor in which we understand *being in a state* metaphorically as *being within a bounded location*. Change of state is understood as movement from one state-location to another. The metaphor here is a conceptual mapping across two different experiential domains, namely, physical locations/motions and abstract states and changes of state (typically either physical states or psychological/emotional states). The underlying metaphor, which we named the Location Event Structure metaphor, consists of the following mapping:

• States Are Locations
• Change of State is Motion From One Location to Another
• Manner of Change is Manner of Motion
• Means of Attaining a Goal are Paths of Motion
• Causes Are Physical Forces
• Causation Is Forced Movement from one Location to Another
• Difficulties are Impediments to Motion
• Purposes are Desired Locations (Destinations)
• Progress Toward Goal is Motion Along a Path

In English (and most other languages around the world), there are hundreds of linguistic expressions that manifest these basic mappings. Thus, we say things like “She’s *in* a funk” (State As Location), “He *went from* good *to* bad” (Change Of State As Movement From One Location To Another), “This is *the way to* happiness” (Means To Goal Is A Path). “Her death *pushed* me *over the edge*” (Causation Is Forced Movement), “He just *stumbled into* the relationship with her” (Manner Of Change Is Manner Of Motion), “Don’t let your fear *keep you from* going where you want to be in life” (Difficulties Are Impediments To Motion), and “I’m *half-way to* paradise” (Progress Toward Goal Is Motion Along A Path).

Notice that the source domain in conceptual metaphors through which we understand the target is nearly always some experience of bodily perception, motion, feeling, personal interaction, or social relations (often those within a family). The mechanism involved is a recruitment of entities, qualities, and structures involved in perception, feeling, bodily movement, etc., to construct understanding of an abstract domain. In our present case of the Location Event Structure metaphor, one of the key structures is what Lakoff (1987) and I (Johnson, 1987) called the Container image schema, which consists of the following minimal structure:

• A boundary
• An interior
• An exterior

As we saw earlier, the Container schema was actually involved in our understanding of the meaning of *cup*, since cups have a three dimensional boundary that defines an interior and exterior.

There are scores of image schemas, such as container, object, source-path-goal, near-far, up-down, center-periphery, right-left, straight-curved, forced motion, degree of intensity, balance, iteration, and on and on (Johnson, 1987; Lakoff, 1987; Hampe and Grady, 2005). Image schemas emerge first in our mundane unreflective bodily engagement with our environment, as recurring patterns of our corporeal/spatial experience. They can be realized in multiple modalities, such as vision, touch, taste, and hearing (Dodge and Lakoff, 2005).

Importantly, these image-schematic structures and relationships are not limited to cases of concrete objects and actions. Their structure and logic can also be appropriated for abstract understanding, such as when, as we just saw above, a State is understood metaphorically as a Location (which is a bounded region, and hence a two- or three-dimensional container). For understanding and reasoning about abstract states, we recruit our knowledge of the source domain, which will have its own corporeal or spatial logic, to draw inferences about the target domain. In the present example, the relevant logic is that of the Container schema, such as:

1. If you are in a container (bounded region), you are not outside of it.
2. If you are outside a bounded region and cross the closed boundary, you will at some point end up within the bounded region (container).
3. If object O is in Container A, and Container A is in Container B, the object O is in Container B.

This image-schematic logic applies, not just to physical containers or bounded spaces, but to abstract containers like states, mathematical sets, institutions, etc. (Johnson, 1987; Lakoff and Nunez, 2000). Thus, if I am *kicked out of* the Beautiful Persons Club, then, by the *embodied logic* of Containment, I know I’m no longer in the club. Or if I use the mathematical metaphor of Sets As Containers, then I get, from (3) above, the logical principle of Transitivity: *All A are B, All B are C, so All A are C*, and this holds for any kind of abstract entity (as metaphorical container) I can imagine within a set-theoretical logic.

So far, the vast majority of research on image schemas and their logics has been based on linguistic and conceptual analysis showing how, in languages all over the world, these basic structures (1) underlie the meanings of terms for physical entities, states, and relations, and (2) shape our understanding of and reasoning, via conceptual metaphor, about abstract concepts. However, in addition to this cognitive linguistic analysis, there is now an emerging theory of the neural structures and processes underlying image schemas (see Dodge and Lakoff, 2005 for a brief summary). There is also a complementary neural treatment of conceptual metaphor (Feldman, 2006; Lakoff, 2008). Lakoff and Srini Narayanan provide the most extensive cognitive linguistic and neural account of both of these phenomena in their forthcoming book, *How Brains Think*.

## Bio-functional Embodied Understanding

Human understanding is profoundly embodied. That is, it is rooted in how our bodies and brains interact with, process, and understand our environments in a way that recruits bodily meaning, neural simulation, and feeling to carry out both concrete and abstract conceptualization and reasoning. “Embodiment” is thus not an add-on to the notion of the bio-functional grounding of human understanding. It is integral to that notion. To say that understanding is bio-functional is necessarily to say that it is embodied, because the relevant functional operations are those of an embodied organism in ongoing engagement with its material, interpersonal, and cultural environments. This perspective overthrows centuries of disembodied views that either deny or overlook the way our bodies, functioning within our changing environments, generate meaning, thought, and, I might add, values (Johnson, 2014).

We obviously do not yet have the full story of embodied understanding—how it is worked up in our formative experience, how it emerges in and is realized through our bodies and brains, how it undergirds and shapes our conceptualization and reasoning, and how it allows us to be “at home” in our world through our bodily engagement with our surroundings. However, we are beginning to develop the tools, techniques, and experimental methods for studying the nature and operation of our bio-functional understanding in sufficient detail to make it clear why we must never again revert to disembodied views of mind, thought, language, and values.

### Conflict of Interest Statement

The author declares that the research was conducted in the absence of any commercial or financial relationships that could be construed as a potential conflict of interest.
